# A Text-book upon the Pathogenic Bacteria

**Published:** 1901-08

**Authors:** 


A Text-book upon the Pathogenic Bacteria.—For students of medicine and physicians. By Joseph McFarland, M. D., Professor of Pathology in the Medico-Chirurgical College, Philadelphia; Pathologist to the Medico-Chirurgical Hospital; Fellow of the College of Physicians of Philadelphia, etc. With 142 illustrations; third edition; revised and enlarged; 621 pages. Price, $3.25. Philadelphia: W. B. Saunders & Co. 1900.

This book presents to the reader a clear account of pathogenic bacteriology. The technic of the subject is well and plainly described, placing a working knowledge within easy reach of the practitioner. The illustrations are ample and the arrangement of subject matter is based upon a long experience, and it is convenient and practical. The author does not intend that the book should be considered a complete laboratory guide, but it is comprehensive and elaborate enough in the consideration of methods to meet the purposes of students and practitioners. It is a valuable work.

